# Concerns about anesthetic standardization and interpretability in the comparison of regional anesthesia with dexmedetomidine versus general anesthesia

**DOI:** 10.1016/j.breast.2026.104855

**Published:** 2026-06-29

**Authors:** Shingo Kawashima, Atsushi Kobayashi, Wakana Kawashima, Masahiko Ohashi, Hiroyuki Kinoshita

**Affiliations:** a Departments of Anaesthesiology and Intensive Care, Hamamatsu University School of Medicine, 1-20-1 Handayama, Chuo-ku, Hamamatsu, Shizuoka, 431-3192, Japan; b Department of Anaesthesiology, Seirei Mikatahara General Hospital, 3453 Mikatahara-cho, Kita-ku, Hamamatsu, Shizuoka, 433-8558, Japan; c Department of Anaesthesiology, Hamamatsu Medical Center, 328 Tomitsuka-cho, Naka-ku, Hamamatsu, Shizuoka, 432-8580, Japan; d Department of Dental Anaesthesiology, Institute of Biomedical Sciences, Tokushima University Graduate School, 3-18-15 Kuramoto-cho, Tokushima, Tokushima, 770-8504, Japan

**Keywords:** Regional anesthesia, Dexmedetomidine, General anesthesia, Anesthetic depth, Local anesthetic systemic toxicity, Block success rate, Quality of recovery (QoR-15)

We read with interest the study by Ling et al. comparing regional anesthesia (RA) with dexmedetomidine (DEX) sedation to general anesthesia (GA) for ambulatory breast surgery [1]. Although the authors conclude that the RA-DEX approach provides superior recovery quality (QoR-15), we have several concerns regarding the clinical standardization of the control group and the interpretability of their findings.

First, the anesthetic regimen in the general anesthesia group appears to introduce a significant methodological bias, precluding a fair comparison between the two techniques. The combination of midazolam, sufentanil, propofol, and continuous dexmedetomidine infusion—administered without any reported method of anesthetic depth monitoring—suggests that patients may have been exposed to unnecessarily deep anesthesia. Such a regimen includes multiple agents with prolonged residual effects, which are known to impair early recovery and increase the risk of postoperative nausea and vomiting (PONV). In our previous work, we demonstrated that the use of regional blocks allows for significant reductions in anesthetic dosing [2], and that ondansetron effectively decreases PONV when combined with block-based conscious sedation [3]. These findings suggest that the poorer recovery in the GA group is not attributable to general anesthesia per se, but rather to the unusually heavy anesthetic regimen used in this study, which likely contributed to both the lower QoR-15 scores and the unexpectedly high PONV rate despite prophylactic granisetron [1]. Thus, the reported superiority of regional anesthesia may reflect the consequences of excessively deep general anesthesia rather than a true advantage of the regional technique.

Second, the regional anesthesia regimen used in this study raises substantial concerns regarding both safety and generalizability. The RA group received a total of 50 ml of 0.3% ropivacaine—a remarkably large volume for breast-conserving surgery and one that approaches thresholds associated with local anesthetic systemic toxicity (LAST). Given that intercostal and pectoral plane blocks are characterized by rapid systemic absorption [4,5], such a high-dose regimen carries a substantial risk of early-peak plasma concentration and subsequent toxicity. Such a high-volume, multi-site block strategy is feasible largely because the study population was relatively young, otherwise healthy, and of normal body habitus; its safety in older or comorbid patients remains uncertain. It remains unclear whether this approach could be safely applied to older patients or those with comorbidities, in whom reduced clearance and increased susceptibility to toxicity would be expected. Moreover, the absence of a standardized postoperative multimodal analgesic regimen (e.g., scheduled acetaminophen) and reliance on rescue analgesia inherently favor the early analgesic window of a high-dose block, potentially exaggerating the apparent superiority of regional anesthesia [6]. Taken together, the block technique and dosing used in this study appear tailored to a highly selected population and cannot be readily generalized to broader clinical practice.

Finally, the further concern is the authors’ report of a 100% success rate for PECS and multilevel intercostal nerve blocks. In clinical practice, achieving a zero-failure rate across 48 consecutive cases is highly unusual given the anatomical variability of the pectoral and intercostal regions. Because the protocol allowed additional local infiltration or conversion to general anesthesia when block efficacy was inadequate, intraoperative supplementation may have been misclassified as “success,” artificially inflating the reported success rate. The study does not define what constituted block success, nor does it describe the frequency of supplemental infiltration, the presence of intraoperative discomfort, or any objective assessment of block adequacy. Without transparent reporting of these elements, it is difficult to determine whether the blocks truly provided complete surgical anesthesia or whether unreported partial failures influenced analgesic requirements and patient-reported outcomes.

In summary, while this study addresses a relevant clinical topic, the observed superiority of regional anesthesia is likely confounded by suboptimal general anesthesia management and a high-volume block protocol that may not be safely generalizable. Without addressing these methodological biases, the conclusion that RA-DEX is inherently superior to GA remains premature.

## Funding

Not applicable.

## CRediT authorship contribution statement

**Shingo Kawashima:** Conceptualization, Methodology, Supervision, Validation, Visualization, Writing – original draft, Writing – review & editing. **Atsushi Kobayashi:** Validation, Writing – review & editing. **Wakana Kawashima:** Conceptualization, Validation, Writing – review & editing. **Masahiko Ohashi:** Validation, Writing – review & editing. **Hiroyuki Kinoshita:** Conceptualization, Supervision, Validation, Writing – original draft, Writing – review & editing.

## Declaration of competing interest

The authors declare that they have no known competing financial interests or personal relationships that could have appeared to influence the work reported in this paper.
